# Enzyme Entrapment in Amphiphilic Myristyl-Phenylalanine Hydrogels

**DOI:** 10.3390/molecules24162884

**Published:** 2019-08-08

**Authors:** Natashya Falcone, Tsuimy Shao, Roomina Rashid, Heinz-Bernhard Kraatz

**Affiliations:** 1 Department of Chemical Engineering and Applied Chemistry, University of Toronto, 200 College Street, Toronto, ON M5S 3E5, Canada; 2 Department of Physical and Environmental Sciences, University of Toronto Scarborough, 1065 Military Trail, Scarborough, ON M1C 1A4, Canada; 3 Department of Chemistry, University of Toronto, 80 St. George Street, Toronto, ON M5S 3H6, Canada

**Keywords:** amino acids, protein, hydrogels, enzyme entrapment

## Abstract

Supramolecular amino acid and peptide hydrogels are functional materials with a wide range of applications, however, their ability to serve as matrices for enzyme entrapment have been rarely explored. Two amino acid conjugates were synthesized and explored for hydrogel formation. These hydrogels were characterized in terms of strength and morphology, and their ability to entrap enzymes while keeping them active and reusable was explored. It was found that the hydrogels were able to successfully entrap two common and significant enzymes—horseradish peroxidase and α-amylase—thus keeping them active and stable, along with inducing recycling capabilities, which has potential to further advance the industrial biotransformation field.

## 1. Introduction

Amino acid and peptide-based supramolecular gels are functional materials that have been the subject of research due to their versatility and their wide range of applications in many fields [1]. Interactions generating the supramolecular network present in physical gels are the result of non-covalent interactions between gelator molecules. Non-covalent interactions present in amino acid and peptide gel materials include hydrogen bonding, van der Waals interactions, π−π stacking, and hydrophobic interactions. Physical gels are also affected by the external environment and are able to undergo sol–gel phase transitions in response to temperature [2], pH [3], and solvent, which allows for tunable properties of these materials. Although supramolecular amino acid and peptide hydrogels formed by self-assembly of amphiphilic molecules have served as scaffolds for tissue engineering and wound healing [4,5], mediums for environmental remediation [6,7], biomaterials for drug delivery, and as antimicrobial agents [8,9,10], their applications as matrices for enzymatic reactions to occur in have been rarely explored.

**Scheme 1:**
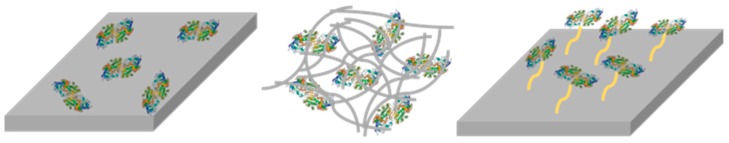
The three enzyme immobilization methods; adsorption, entrapment, and covalent bonding (from left to right).

Enzymes are a class of proteins that exhibit high efficiency and specificity for catalyzing many reactions in our bodies and in biochemical and chemical reactions. In industrial biotransformations, using enzymes as biocatalysts are attractive as they possess high selectivity, run under milder and greener reaction conditions, and eliminate the need of expensive and toxic metal catalysts [11]. Enzymes are attractive “green” alternatives to chemical catalysts within the industrial sector, but their robustness to environmental conditions needs optimizing. The issue with adopting enzymes as biocatalysts on the industrial scale also includes their low stability and poor recycling ability. However, it has been reported that immobilized enzymes have a greater stability compared to free enzymes. There are three main immobilization methods: Adsorption [12], entrapment [13], and covalent bonding (Scheme 1) [14]. While all have pros and cons, entrapment, meaning the enzyme remains within the gel matrix, has been more readily explored due to adsorption being weak and covalent bonding resulting in binding of the active sites of enzymes. Most reports of enzyme immobilization using the entrapment method commonly use silica or polymer matrices [15,16,17,18,19,20,21,22]. Chen et al. encapsulated triacylglycerol lipase within biomimetic silica through the catalysis of polyallylamine and found encapsulation not only stabilized the lipase but also enhanced the activity [16]. A *N*,*N*’-methylenebisacrylamide (MBA)-cross-linked *N*-isopropylacrylamide (NIPAAm) hydrogel was also used to form a hemin-based mimetic enzyme with a higher activity compared to hemin alone [23]. In addition, numerous applications of alginate gel matrices have been reported in enzyme entrapment studies [24,25].

Entrapment in an amino acid or peptide supramolecular hydrogels are rarely reported but has the potential to be one of the leading matrices for keeping the enzymes stable and active in an aqueous, physiological material [26]. It also has the potential to be an improved matrix compared to polymeric or alginate entrapment matrices due to the common characteristics peptide hydrogels have with enzymes. These include amino acids as the main building block, supramolecular interactions as the driving force for self-assembly, amphiphilicity of the structures, and a supramolecular structure as the result of the formation of a network of nanofibers [26]. Park et al. used a diphenylalanine hydrogel to immobilize a glucose oxidase enzyme for employment of a biosensing platform [27]. Xu et al. reported a molecular peptide hydrogel-immobilized enzyme exhibiting supractivity and high stability [20]. In addition, this group showed a supramolecular-hydrogel-encapsulated hemin as an artificial enzyme to mimic peroxidase and exhibited self-immobilization of a phosphatase enzyme in a gel for high activity and stability [26,28,29,30,31]. Miller et al. also reported enzyme entrapment within a hydrogel matrix and described how molecular hydrogels are ideal natural materials for the support of enzymes as they reversibly self-assemble under aqueous conditions at physiological temperatures [32]. However, to avoid enzyme leaching they had to covalently attach an enzyme to a β-sheet self-assembling peptide. Since then peptide hydrogels have been rarely explored as an entrapment matrix compared to silica or polymer matrices. To the best of our knowledge there are no recent (<five years) references of amino acid and peptide hydrogels for enzyme entrapment, but many reported for silica alginate, or polymeric matrices. For these reasons the uses of amino acid and peptide hydrogels specifically, as a matrix is significant as they possess many advantages including biocompatibility and biodegradability, in addition the amphiphilic nature of the peptide nanofibers is also reported to facilitate the transportation of the substrate and product through the hydrogel before and after catalytic transformations [32].

In this article, we expand the uses of amphiphilic-amino acid hydrogels for one of the enzyme immobilization methods as an entrapment matrix for potential improvement to their stability, protection from degradation, and increase in the ability of enzyme recycling over longer periods of time. Two amphiphilic amino acid conjugates composed of an aliphatic chain from myristic acid and l- and d-Phenylalanine (Phe) have been synthesized and explored for hydrogel formation. Previously, our group reported only the L enantiomer of the compound in a 0.1 M phosphate buffer solution at pH 7.25–8.5 and looked at metal binding studies [33]. Here, both enantiomeric compounds formed a hydrogel in a 50 mM monobasic phosphate buffer solution at physiological pH 7 and their full characterization including their strengths through rheology experiments and morphology through transmission electron microscopy (TEM) studies was compared. Interestingly, it is also shown in the CD spectrum that the Phe compound was found to form an ordered structure exhibiting a distinct ultra-narrow peak around 220–230 nm, which attributes to the formation of Phe-based chiral structures that correspond to structural aggregates.

**Scheme 2:**
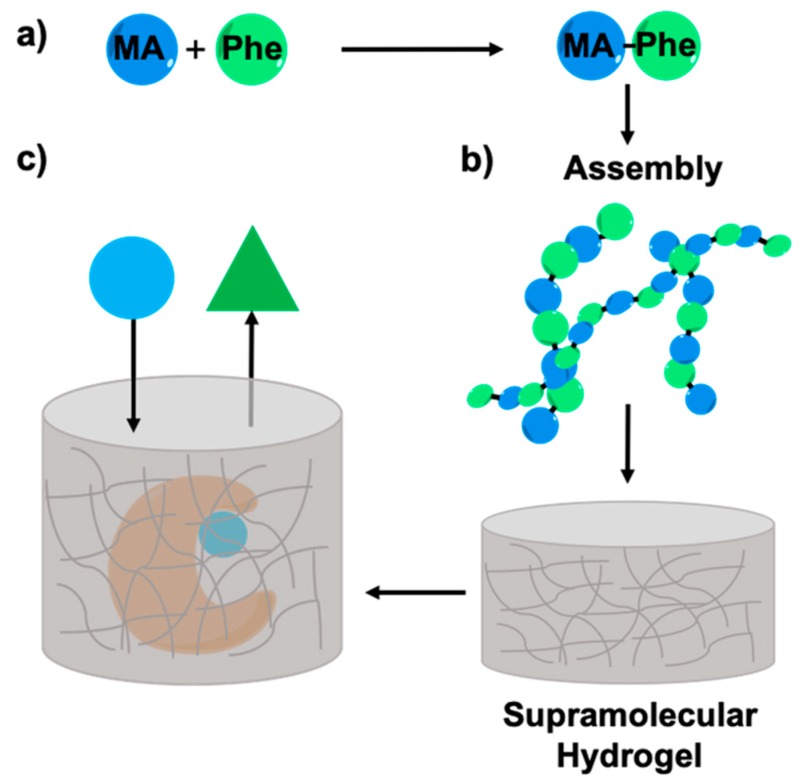
Scheme summarizing the overall research work. (**a**) Synthesis of amino acid conjugates using Myristic acid (MA) and Phenylalanine (Phe); (**b**) formation of hydrogel through supramolecular assembly of amino acid conjugates; (**c**) enzyme entrapped in supramolecular hydrogel, substrate (blue circle) diffusing in and product (green triangle) diffusing out.

Both hydrogels were tested for entrapment capabilities with two common and significant enzymes: Horseradish peroxidase (HRP) and α-amylase. Product quantification and recycling experiments were performed, and it was found that the enzymes immobilized in the gels were stable and active for one month and were able to catalyze multiple reactions over a one-week time period with minimal leaching. Scheme 2 summarizes the work in this report. Here, we present a qualitative evaluation rather than a quantitative kinetic evaluation at present to stay within the scope. The synthesized hydrogels did not deactivate the enzyme and were found to support both enzymes to perform catalytic reactions through the matrix.

## 2. Results

### 2.1. Synthesis of Myristic Acid-l/d-Phenylalanine (MA-l/d-Phe-OH)

**Figure 1:**
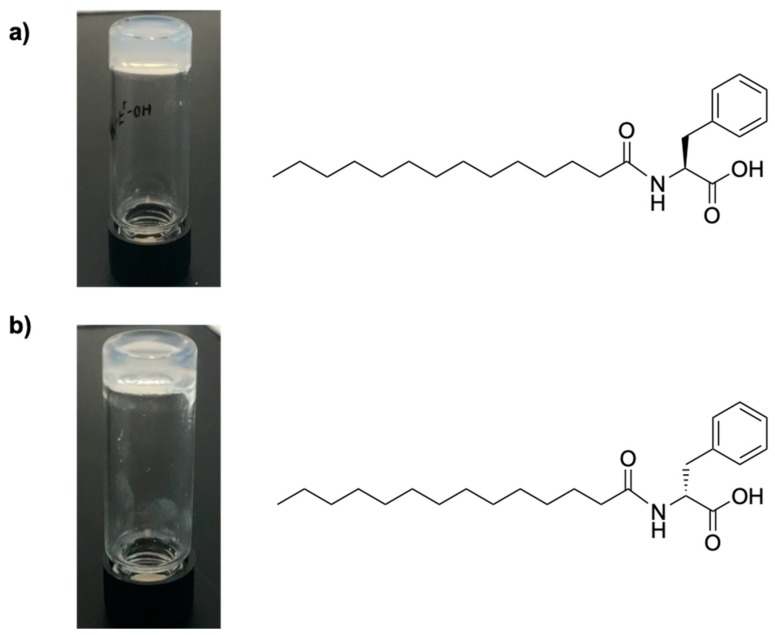
(**a**) Aliphatic-amino acid hydrogel shown through an inverted vial test and the molecular structure of MA-l-Phe-OH; and (**b**) aliphatic-amino acid hydrogel shown through an inverted vial test and the molecular structure of MA-d-Phe-OH.

MA-l/d-Phe-OH was synthesized using standard carbodiimide coupling procedures using myristic acid and the amino acid phenylalanine, in the presence of HOBt [34]. Further synthetic details can be found in the experimental section. Chemical structures of the amino acid conjugates are shown in Figure 1 and their full characterization can be found in the Supporting Information.

The self-assembling behavior of the conjugates was explored in a 50 mM monobasic phosphate buffer solution at pH 7. When a hot solution of the compound cooled to room temperature, a viscoelastic gel was obtained. Surprisingly, this compound had a very low MGC, meaning only a small amount of compound is required to form the hydrogel material, thus making it cost- and material-efficient. At this MGC however, the melting temperature of the gel is low at around 37 °C, at which the gel turns into a liquid state. As we increase the concentration of gelator, the melting temperature of the gel increases as more heat is required to break the increased amount of interactions between more gelator molecules, therefore at our working concentration of 1% the T_gel_ was 51 °C. The tan δ values will be discussed in the following section. The gelation information for the compounds including the minimum gelation concentration (MGC), melting temperature of the gel (T_gel_) and the tan δ values at 1% is reported in Table 1.

### 2.2. Rheology Studies

**Figure 2:**
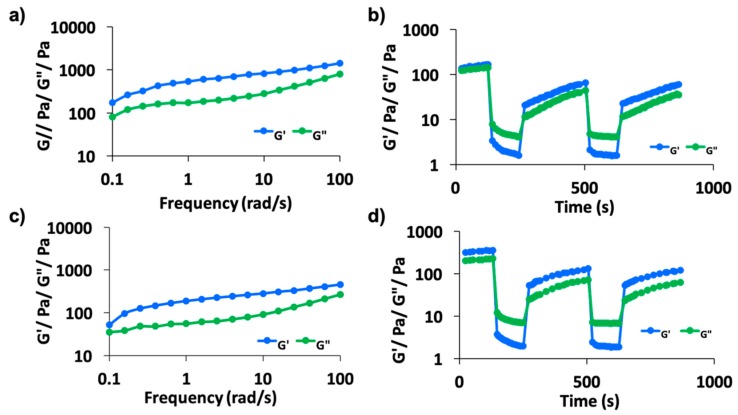
(**a**) Frequency sweep experiments on MA-l-Phe-OH. G′ (storage modulus) remains higher than G″ (loss modulus). (**b**) Step strain experiments of MA-l-Phe-OH. Cycle 1 under 0.1% straight and G′ remains higher than G″, cycle 2 under 100% strain gel is reverted to the sol phase where G″ is higher than G′, cycle 3 lifts the strain and is back to 0.1% and is back in gel phase as G′ is greater than G″. This is repeated for two more cycles to show self-healing properties of the gel. (**c**) Frequency sweep experiments on MA-d-Phe-OH. G′ (storage modulus) remains higher than G″ (loss modulus). (**d**) Step strain experiments of MA-d-Phe-OH. Cycle 1 under 0.1% strain and G′ remains higher than G″, cycle 2 under 100% strain gel is reverted to the sol phase where G″ is higher than G′, cycle 3 lifts the strain and is back to 0.1% and is back in gel phase as G′ is greater than G″. This is repeated for two more cycles to show self-healing properties of the gel.

Frequency sweep rheology experiments were performed to study the mechanical strength of the gels (Figure 2). In oscillatory shear measurements, the shear storage modulus, G′, loss modulus, G″, and loss factor, tan δ, are critical hydrogel properties monitored against time, frequency, and strain. The complex modulus of the tested material is G*(w) = G′ + iG″ as the real (elastic or in phase) and imaginary (viscous or loss or out of phase) components of G* [35]. For a typical viscoelastic gel, the storage modulus, G′, remains higher than the loss modulus, G″ [35,36]. G′ measures the deformation energy stored during the shear process of a test material (i.e., stiffness of the material) and G″ is representative of the energy dissipated during shear (i.e., the flow or liquid-like response of the material) [35]. A piece of the 1% hydrogel material was placed under the rheometer geometry and frequency sweep experiments were performed at 0.1% strain from a frequency range of 0.1–100 rad/s. From the data shown in Figure 2a,c, both hydrogels proved to be a viscoelastic gel material as the G′ was greater than G″. The elastic modulus, E′, was estimated as E′ = 2G′(1 + ν), where G′ is the storage modulus measured by the rheometer, ν is Poisson’s ratio assumed to be 0.5 [37], for M-F(L) and (D) E′ was roughly found to be ~725 and 715 Pa. The tan δ value (G″/G′) is a representative of gel elasticity that measures the energy loss owing to viscous flow compared to the energy stored. When this value is greater than 1 the sample behaves as a viscous liquid; conversely, when the value is less than 1 the sample behaves more as an elastic solid [35,36]. The tan δ values for both compounds are reported in Table 1 and it was found that the L and D enantiomer compounds had the same tan δ value of 0.41. The compounds also demonstrated shear-thinning properties. 

Step-strain experiments using the rheometer demonstrating thixotropic properties were also performed in which strain was applied to the gel causing the gel to revert to the solution phase. Thixotropic properties of the gel demonstrate that under normal conditions, the property is a gel and under stressed conditions such as heat, force, or sonication, it becomes less viscous, however, when the stress is removed the gel-state reforms. Self-healing gels that can restore their functionalities and structures after damage have been developed as ‘smart’ soft materials [38]. Rheological studies show the behavior of the gel under mechanical stress and the variation of the storage and loss modulus have been studied under the application and release of shear stress. Stress causes the gel to break, while release of the mechanical stress allows reformation of the hydrogel [39]. In the first cycle in which 0.1% strain was applied, G′ is greater than G″ confirming gel formation, whereas in cycle 2, 100% strain is applied and G″ is found to be greater than G′, thus reverting it to sol state. Strain was removed and set back to 0.1% in the third and fifth cycles to see if the gel reformed, interestingly, both compounds exhibited thixotropic properties shown in Figure 2b,d.

### 2.3. Circular Dichroism

**Figure 3:**
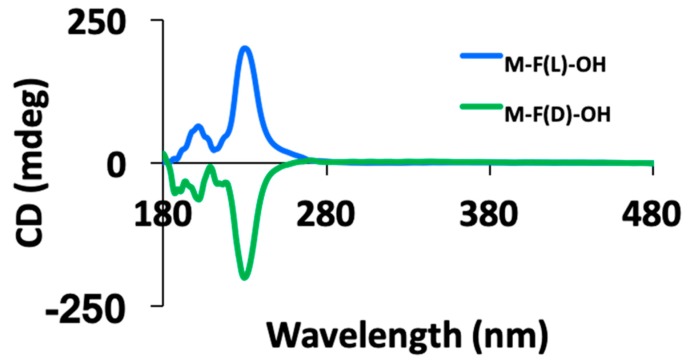
Circular dichroism (CD) spectra of MA-l-Phe-OH in blue and MA-d-Phe-OH in green showing both enantiomers being mirror images of each other in 1% gel state. The spectra also demonstrate a narrow peak and the self-assembling characteristics of phenylalanine as reported in the literature.

As amino acids represent one of the simplest classes of small biological molecules that are also chiral, they were the first molecules to be characterized by circular dichroism (CD) [40]. To further investigate the supramolecular structure and the presence of both enantiomers, CD spectroscopy was performed. CD spectroscopy has been reported to be a valuable technique that can be used to determine the molecular chiral arrangements in a self-assembled state [41,42]. Bands below 300 nm arise from chiral amino acids, while L amino acids exhibit a broad positive CD peak in the 200–207 nm region. Typical CD peaks for amino acids result from the carbonyl bond at 190–200 nm representing the π-π* transition and at 210–230 nm representing the n-π* [40]. One of the most well-known properties of aromatic moieties, such as the phenyl ring in the amino acid phenylalanine (Phe), is their ability to aggregate/stack through π−π interactions. Although single amino acids are not known to form secondary structures, the amino acid phenylalanine has been highlighted in literature as a special case as it resembles the self-assembling process of the diphenylalanine compound [40]. Phe was found to form an ordered structure exhibiting an ultra-narrow peak around 220–230 nm, which attributes to the formation of Phe-based chiral structures that correspond to structural aggregates [40]. As shown in Figure 3, the presence of chiral amino acids and the interesting characteristic of the self-assembling Phe is highlighted and observed as a major peak at around 225 nm for the l-enantiomer is shown, while the d-amino acid induced the opposite helicity, revealing that amino acid chirality is opposite for each enantiomeric pair. Smaller peaks are observed around 200 nm indicating the carbonyl bond, which, as mentioned, is typical of amino acids.

### 2.4. Morphological Investigation

**Figure 4:**
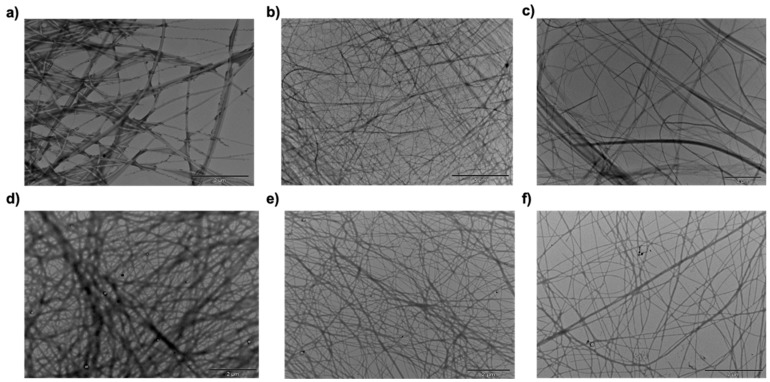
TEM images of MA-l-Phe-OH and MA-d-Phe-OH. (**a**–**c**) Three-dimensional fibrous network of MA-l-Phe-OH, and (**d**–**f**) three-dimensional fibrous network of MA-d-Phe-OH. The scale bar is 2 μm for all images except (**b**), which is 5 μm.

To investigate the morphological features of the self-assembled amino acid conjugates, transmission electron microscopy (TEM) studies were performed. The Formvar carbon coated copper TEM grid was dipped into the hydrogel, followed by drying at room temperature and under vacuum. The grid was then directly put on the TEM instrument mentioned in the experimental section. The morphology of a nanofiber matrix is reported for both L and D (Figure 4), where we observed long fibers, which showed a three-dimensional interweaving architecture. Fibril structures have been reported as the main morphology of strong peptide based-gel materials [43,44,45,46]. The fibers had an average thickness of 0.056 ± 0.011 μm for MA-l-Phe-OH and 0.058 ± 0.021 μm for MA-d-Phe-OH, which is similar to reported amino acid and peptide hydrogels. A histogram of the fiber thickness for both compounds is found in Figure S9. Additional TEM images of the hydrogels with entrapped enzyme can be seen in Figure S10. A three-dimensional fibrous network is also observed with additional black particles within the fibers, one may speculate this small difference may be due to the entrapped enzyme.

### 2.5. Enzyme Entrapment

**Scheme 3:**
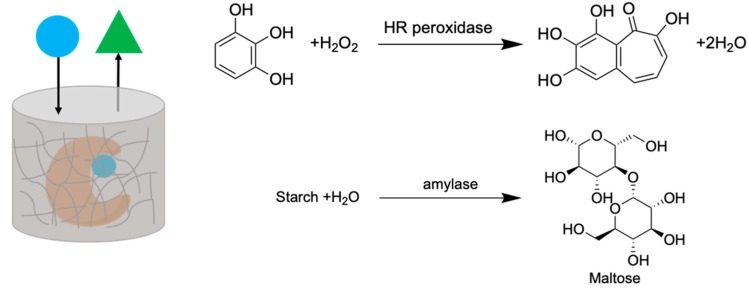
Enzyme reaction schemes of horseradish peroxidase (HRP) and amylase enzymes. HRP converts pyrogallol (substrate) to purpurogallin (product) and amylase converts starch (substrate) to maltose (product).

To investigate the ability of our amino-acid hydrogels to act as a matrix for enzyme entrapment, 50 μL of a 1 mg/20 mL enzyme solution of both horseradish peroxidase (HRP) and α-amylase were added to the hydrogels. Scheme 3 shows the reactions both enzymes catalyze. Specifically, this was done by heating the compound in 50 mM monobasic phosphate buffer pH 7 until a clear solution was obtained. Once the temperature of the vial cooled, 50 μL of the enzyme solution was added into the gel solution. As the solution further cools to room temperature on the bench top, the compound forms a hydrogel with the enzyme entrapped inside. With the addition of the enzymes, the resulting hydrogels were determined to have similar viscoelastic properties as the tan δ values remained the same for both L and D gels with both enzymes (Table 1). The rheology data for the gels with entrapped enzymes can be seen in Figure S11. 

**Figure 5:**
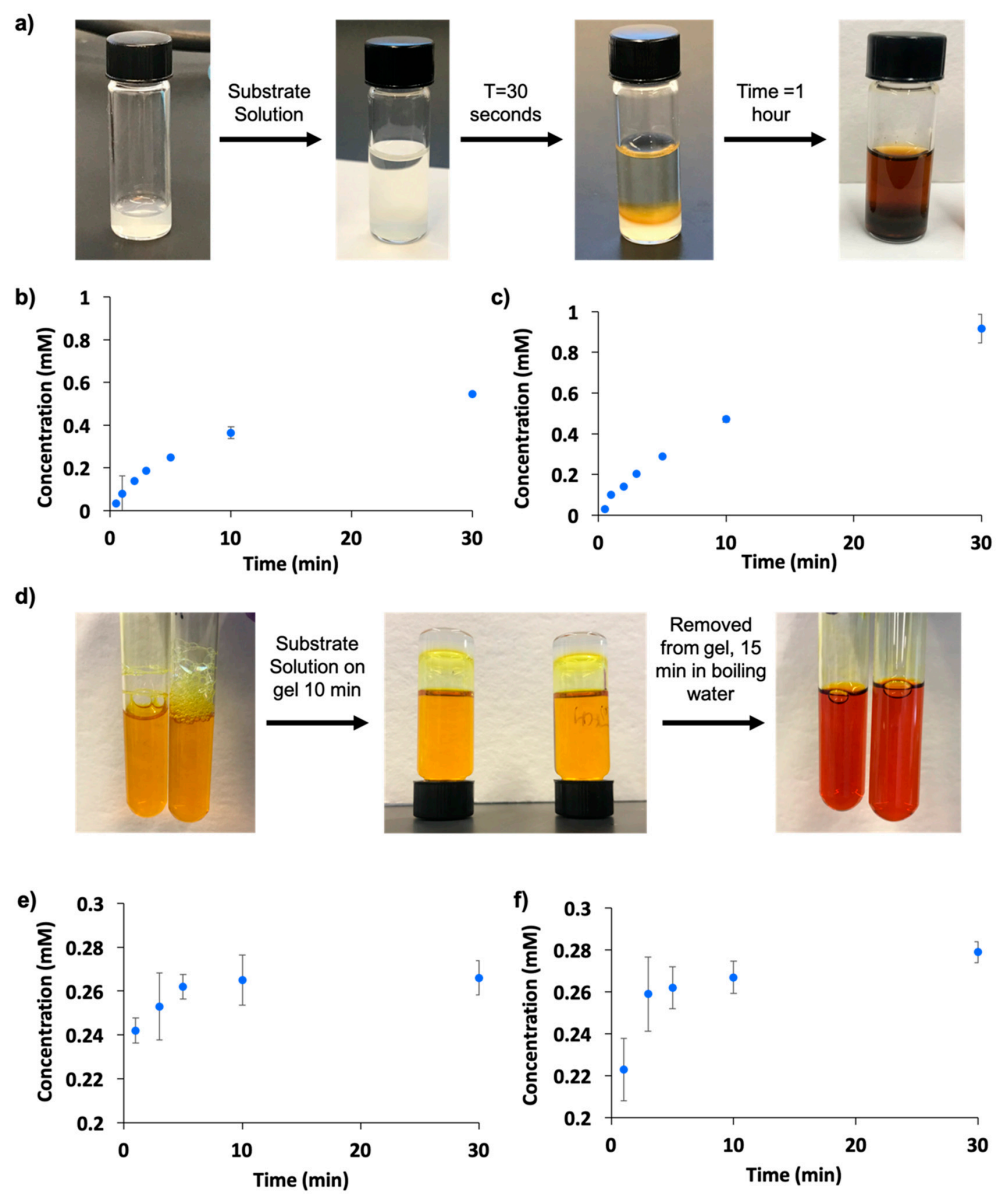
(**a**) Image of the amino acid hydrogels with HRP entrapped prior to substrate solution addition on top and after three minutes. (**b**) Quantification data of product formation using MA-l-Phe-OH entrapped hydrogel over time using absorbance at 420 nm and the known extinction coefficient of 2640 cm^−1^ M^−1^. (**c**) Quantification data of product formation using MA-d-Phe-OH entrapped hydrogel over time using absorbance at 420 nm and the known extinction coefficient of 2640 cm^−1^ M^−1^. (**d**) Image of the amylase-entrapped amino acid with the substrate solution shown prior to addition on top of hydrogel and after 10 min on top of the hydrogel and 15 min. in boiling water. When in contact of a reducing sugar, the color reagent containing dinitrosalicylic acid goes from a yellow-orange color to a darker orange-red color. (**e**) Quantification data of product formation using MA-l-Phe-OH entrapped hydrogel over time using absorbance at 540 nm and the calculated extinction coefficient of 1340.6 cm^−1^ M^−1^. (**f**) Quantification data of product formation using MA-d-Phe-OH entrapped hydrogel overtime using absorbance at 540 nm and the calculated extinction coefficient of 1340.6 cm^−1^ M^−1^.

The substrate solution containing 2.6 mL of 50 mM monobasic phosphate buffer pH 7, 0.16 mL of a 30% H_2_O_2_ solution, and 0.32 mL of a 50 mg/mL pyrogallol solution was added on top of the enzyme-entrapped hydrogel. Within seconds, a color change was observed in the substrate solution indicating the formation of product, purpurogallin, which could be quantified using ultraviolet (UV) spectroscopy at 420 nm and its extinction coefficient of 2640 M^−1^ cm^−1^ in 50 mM phosphate buffer at pH 7. In Figure 5a, the color change of the substrate solution after 30 s was observed, indicating that the enzyme was very active in our hydrogel. The color change was first noticed at the surface of the gel and as time increases the color change throughout the whole gel was observed. This is due to the initial contact of the substrate and enzyme on the hydrogel surface, however as the substrate diffused in the gel and reacted with more enzyme, more product was formed causing the full gel to turn the brown color as seen in Figure 5a after one hour. The formation of product was then quantified over a period of time as shown in Figure 5b,c. It is observed that as time increases, giving the substrate time to diffuse into the gel and react with the entrapped enzyme, the formation of product also increased. This follows a similar trend to the enzyme in solution, where as time increased, product formation also increased. However, due to the instant and direct contact of the enzyme with the substrate in solution, product formation plateaus much quicker. The activity of the enzyme in solution can be found in Figure S12.

The substrate solution for the amylase enzyme, consisting of 1 mL of a 100 mg/10 mL solution of starch, 1 mL of 50 mM monobasic phosphate buffer pH 7, and 1 mL color reagent (dinitrosalicylic acid), was placed on top of the hydrogel containing the amylase enzyme. Since maltose cannot be directly quantified, a color reagent solution consisting of dinitrosalicylic acid was used as it is a well-known method for quantification of reducing sugars [47,48]. In the presence of reducing sugars, such as the product of this reaction (maltose), the yellow solution turns red. With a standard curve of maltose and color reagent (Figure S13), the amount of product produced from the enzymatic reaction was quantified. Figure 5d shows the substrate solution with a color reagent before being placed on top of the enzyme-entrapped hydrogel, left for 10 min on top of the hydrogel, and then followed by 15 min in boiling water after which the substrate solution turned red. This proves again that our enzyme was active in the hydrogel as product formation is observed. The product was quantified using the extinction coefficient found from the standard curve shown in Figure S13 and in Figure 5e,f, an increase in product formation can also be seen over time. 

### 2.6. Enzyme Leaching

When testing for enzyme entrapment it is important to look at enzyme leaching to ensure all of the enzyme is not leaching out and catalyzing the reaction in solution on top of the gel, but that the substrate is diffusing through the porous gel and reacting with the entrapped enzyme. In almost all cases of entrapment, enzymes may leach out of the gel. It has been reported however, due to the non-covalent forces between the amino-acid conjugates involved in supramolecular gelation, and the same forces involved in an enzyme’s supramolecular structure, that these forces help stabilize most of the enzymes in the gel [23]. To prove that the activity reported from the enzymes in the gel was not solely due to enzymes leaching out and catalyzing the reaction in the solution on top of the gel, leaching experiments were performed. These experiments involved placing only a phosphate buffer on top of the gels with entrapped enzymes and taking aliquots of the buffer at different time points. The aliquots were then added to the substrate solutions (same amounts used when testing the activity of enzymes in the gel) and activity was measured. Further experimental detail can be found in the experimental section. Figure S14 shows a comparison of activity of the product formation when catalyzed on the gel with entrapped enzyme to the activity of product formation using the leached-out enzyme in the buffer. It is clearly shown that although minimal enzymes are leaching and catalyzing the reaction slowly, it does not compare to the amount of product formed from the catalytic reaction through the gel. In addition, Figure S15 shows an image of two sets of L and D hydrogels, one contains the enzyme inside the gel while the other does not, the enzyme is added later to the substrate solution on top. Here, we can visually see that the substrate is being catalyzed in the gel as a brown color developed for the hydrogels with enzyme entrapped, while no color change is observed for the gel with no enzyme entrapped but rather just on top with the substrate solution. For both enzymes in both L and D hydrogels, one can conclude that the activity is not solely coming from enzyme leaching out and catalyzing the reaction in solution, but in fact the catalytic reaction is occurring through the gel matrix with the majority of enzyme entrapped in the fibers. 

### 2.7. Enzyme Recycling and Stability

**Figure 6:**
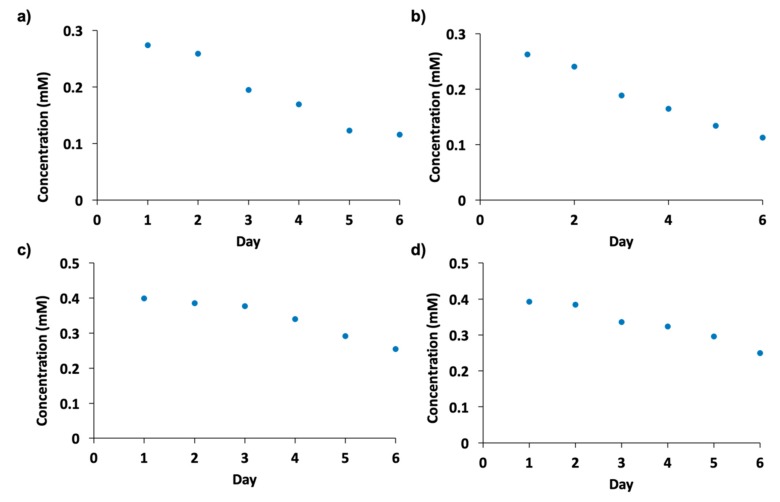
Recycling experiments with both enzymes entrapped in L and D amino acid hydrogels. (**a**) MA-l-Phe-OH hydrogel with an HRP enzyme entrapped. Fresh substrate solution was placed on top of the gel each day and product was quantified at absorbance 420 nm. (**b**) MA-d-Phe-OH hydrogel with HRP enzyme entrapped. A fresh substrate solution was placed on top of the gel each day and product was quantified at absorbance 420 nm. (**c**) MA-l-Phe-OH hydrogel with amylase enzyme entrapped. Fresh substrate solution was placed on top of the gel each day and product was quantified at absorbance 540 nm. (**d**) MA-d-Phe-OH hydrogel with an amylase enzyme entrapped. Fresh substrate solution was placed on top of the gel each day and the product was quantified at absorbance 540 nm.

Reported advantages of enzyme entrapment in amino acid and peptide hydrogels are to increase its stability over longer periods and to be able to reuse and recycle the same enzyme for many catalytic reactions [26]. A recycling experiment was performed where the enzyme-entrapped hydrogels catalyzed a fresh substrate solution every day for one week. Each day the substrate solution was removed, and a fresh solution was placed on top of the hydrogel to see if the enzymes were still active and could catalyze multiple reactions over the one-week period of time. Figure 6a–d shows the activity after 10 min in the enzyme-entrapped hydrogels every day over a one-week period. Over time less product was produced, which was expected; however, the enzyme was able to remain active and be re-used multiple times in the hydrogel. Here, the same entrapped enzyme was able to catalyze multiple catalytic reactions without the usual requirement of a new catalyst each day. 

In addition to being able to recycle and reuse the enzyme over time, a preliminary long-term stability test of the enzyme was performed. Here, the enzymes were placed in both L and D hydrogels and were left alone for one month. After this time, the activity of the enzyme was tested by placing fresh substrate solution on top and quantifying the solution after 10 min. Figure S16 shows the comparison of enzyme activity after 10 min on the gel on the first day to the 30th day. Although less active, it was very interesting to see both enzymes still being able to catalyze both enzymatic reactions over this long time period. 

## 3. Discussion

In this study two amino-acid hydrogels were synthesized, characterized, and tested for their ability to serve as a matrix for enzyme entrapment. Firstly, these hydrogels showed exceptional stability on their own. They are able to remain in hydrogel state for over a year if no external factors are involved which itself proves to be a remarkable biomaterial. The hydrogels exhibit thixotropic properties where upon addition and removal of strain, the gel material breaks and reforms the hydrogel state. They also have optimal three-dimensional fiber morphology where the nanofibers and amphiphilic surfaces in the hydrogels allow rapid mass transfer as well as an optimal network to keep the enzyme entrapped [26]. In addition, the materials show the interesting self-assembling properties of Phe and the presence of both enantiomers on CD spectroscopy. Phe was found to form an ordered structure exhibiting a distinct ultra-narrow peak around 220–230 nm, which attributes to the formation of Phe-based chiral structures that correspond to structural aggregates [40]. These materials can be tailored by changing the concentration of the gel: A more solid-like material is formed when the hydrogel concentration is 5% *w/v* where the material is stiff and less viscous while a viscous, malleable material is formed when the hydrogel is at its MGC. A working concentration of 1% was chosen for all studies as there is a 30-minute wait period after heating the solution before reaching a hydrogel state. This provides optimal time to add in the enzyme solution without deactivating it, thereby allowing the gel to further cool afterwards into a hydrogel with entrapped enzyme. 

Both hydrogels proved to be an optimal matrix for enzyme entrapment as enzymes remained active in the hydrogel, meaning the enzyme did not completely deactivate and can catalyze the biochemical reaction while it was entrapped in the gel. To test the ability of the hydrogels to act as a suitable matrix, the initial activity of both enzymes in the gels were quantified using UV spectroscopy and their respective extinction coefficients. As time increases with the substrate solution on the gel, an increase in product formation is observed as there was more time for substrate diffusion and interaction with the entrapped enzyme. It is also observed that the enzymes do not prefer one enantiomer over the other, as a very similar activity and quantification results were concluded for both L and D hydrogels. Enzyme leaching was also explored, and it was found that there was minimal leakage of the enzyme into the solution on top of the hydrogel. The reported activity using the enzyme that leached out of the gel into the buffer solution was substantially lower compared to the activity of the enzyme entrapped in the gel. This reinstates the idea that the interactions between the amino acid conjugates and the enzyme help to keep the enzyme entrapped, meaning the enzyme stayed within the hydrogel matrix. In addition, preliminary studies show that the same amount of enzyme catalyst entrapped in the gel can catalyze fresh substrate over multiple days, allowing the biocatalyst to be reused, which is beneficial in industrial biotransformations. Future studies involving testing the kinetics and comparing multiple entrapped enzymes are currently ongoing.

A large number of enzymes trapped within sol–gel matrices show that they usually retain their catalytic activity while being protected against degradation [49]. Examples of entrapped enzymes were reported to exhibit better stability, activity, and longer lifetimes than free enzymes, meaning the enzyme’s ability to catalyze the chemical reaction lasts longer, and converts more product while not deactivating from external factors such as heat. For example, Xu demonstrated that hydrogelation provides a useful approach for immobilizing thermally unstable enzymes to significantly enhance their activity and stability (including thermal and operative) of the enzymes [29]. Compared to reported examples of enzyme entrapment, these amino acid hydrogels have similar properties where the stability is prolonged and the enzyme is able to be recycled. However, the number of examples of amino acid and peptide hydrogels as a matrix for entrapment is limited and should be explored further. Due to the fact that amino acid and peptide hydrogels resemble extracellular matrices, as reported in literature [4,5], the enzymes are also able to function in a material that is more biocompatible and biodegradable. In addition, it was reported that unlike the cross-linked random networks in a conventional polymeric hydrogel, the larger diffused channels and amphiphilic surfaces in the molecular hydrogel allow rapid mass transfer of the reactant and the product [29]. The use of amino acid and peptide hydrogels holds great potential to be widely exploited as a matrix for enzyme entrapment. By looking at various amino acid and peptide conjugates with different enzymes and varying concentrations of hydrogels to affect the pore-sizes or entrapment ability for different sizes of enzymes, this leads to a potential variety of gels that can be tailored for different catalytic reactions. 

## 4. Materials and Methods 

Methyl esters of amino acids were purchased form Advanced ChemTech, Louisville, KY, US. Myristic acid, amylase, pyrogallol, starch, and horseradish peroxidase were purchased from Sigma Aldrich, Mississauga, ON, Canada. Chemicals were used without further purification.

Both compounds were synthesized using the standard 1-ethyl3-(3’-dimethylaminopropyl)carbodiimide (EDC)/1-hydroxybenzotriazole (HOBt) method. Myrsitic acid (8 mmol) was dissolved in DCM and HOBt (4 mmol, 0.678 g) and EDC (4 mmol, 0.767 g) were added to the reaction mixture at 0 °C and the reaction mixture was stirred for 30 min in ice. Five mmol of l- or d-Phe-OH was added to the solution and was stirred for 24 h at r.t. Afterwards, a standard aqueous workup was done consisting of sequential washing with aqueous 10% citric acid solution, saturated aqueous NaHCO_3_, and saturated brine solution. This was followed by collection and drying of the organic layer. The crude material was then purified by column chromatography on silica gel using hexane/ethyl acetate solvent system to afford the pure product as a white solid. The compound was then hydrolyzed to remove the OMe protecting group by adding 1.3 mmol (0.5 g) of myristyl-phenylalanine-OMe to a round bottom flask with 16 mmol (0.65 g) of NaOH along with 20 mL of 1,4-dioxane and 20 mL of dH_2_O. The reaction was stirred at room temperature overnight. After the stirring, concentrated HCl was added to precipitate the solution and acidify it to pH 2–3. This was followed by two washes with ethyl acetate. The ethyl acetate layers were collected, dried with anhydrous sodium sulfate, filtered, and evaporated under pressure. This resulted in the pure white powder compound used for gelation.

MA-l-Phe-OH ^1^H-NMR (500 MHz, [D] chloroform): 12.70 (s, 1H, OH), 7.32 (m, 3H; H-Phe), 7.20 (m, 2H; H-Phe), 5.82 (d, 1H; amide NH), 4.86 (td, 1H; CHα), 3.28 (dd, 1H; CH2β), 3.16 (dd, 1H; CH2β), 2.20 (td, 2H; (CH2), 1.58 (td, 2H; (CH2)), 1.27 (m, 20H; (CH2)), 0.90 (t, 9H; (CH3)); ^13^C-NMR (125 MHz, [D6] dimethyl sulfoxide): 173.17 (1C; COOH), 172.09 (1C; C=O) 137.75 (1C; benzene), 129.01 (2C; benzene), 128.00 (2C; benzene), 126.22 (1C; benzene), 53.19 (1C; α-CH), 36.75 (1C; β-CH2), 22.09–35.06 (11C; CH2)), 13.90 (1C; (CH3)); FTIR: 3300 cm^−1^ (amide N-H), 1705 cm^−1^ (ester C=O), cm^−1^ (amide C=O); ESI-MS: *m/z*: 375.2773 [M]; Found *m/z* 376.2851 ([C23 H37 N O3]+H)+; elemental analysis for C23H37NO3: C 73.56, H 9.93; N 3.73; found: C 73.85, H 9.46, N 3.67.

MA-d-Phe-OH ^1^H-NMR (500 MHz, [D] chloroform): 12.71 (s, 1H, OH), 7.32 (m, 3H; H-Phe), 7.19 (m, 2H; H-Phe), 5.85 (d, 1H; amide NH), 4.87 (td, 1H; CHα), 3.28 (dd, 1H; CH2β), 3.16 (dd, 1H; CH2β), 2.20 (td, 2H; (CH2), 1.57 (td, 2H; (CH2)), 1.28 (m, 20H; (CH2)), 0.90 (t, 9H; (CH3)); ^13^C-NMR (125 MHz, [D6] dimethyl sulfoxide): 173.18 (1C; COOH), 172.09 (1C; C=O) 137.75 (1C; benzene), 129.01 (2C; benzene), 128.01 (2C; benzene), 126.23 (1C; benzene), 53.19 (1C; α-CH), 36.75 (1C; β-CH2), 22.09–35.06 (11C; CH2)), 13.90 (1C; (CH3)); FTIR: 3278 cm^−1^ (amide N-H), 1722 cm^−1^ (ester C=O), 1649 cm^−1^ (amide C=O); ESI-MS: *m/z*: 375.2773 [M]; Found *m/z* 376.2846 ([C23 H37 N O3]+H)+; elemental analysis for C23H37NO3: C 73.56, H 9.93; N 3.73; found: C 73.75, H 9.82, N 3.83.

### 4.1. Nuclear Magnetic Resonance (NMR) Spectroscoopy

Compounds were characterized using NMR spectroscopy on a Bruker 500 MHz spectrometer. The sample was dissolved in [D6] dimethyl-sulfoxide at a concentration of 2 mm. Spectra are shown in the Supporting Information.

### 4.2. Fourier Transform—Infrrared Spectroscopy

FTIR spectra of the compound were recorded in the range 3400–400 cm^−1^ using a Bruker Alpha FTIR spectrometer equipped with a diamond for attenuated total reflection. A wet viscous sample was prepared by dissolving both compounds in the phosphate buffer, forming the gel, then keeping the samples under the vacuum to dry.

### 4.3. Transmission Electron Miscroscopy (TEM)

The morphology of the self-assembled compounds was studied using TEM. The sample was prepared by dipping our grid into the viscous hydrogel material on a copper TEM grid (300 mesh size), donated by FORTA Science, coated with Formvar and a carbon film. The grid was allowed to dry by slow evaporation in air and then further dried in a vacuum for 24 h. Images were acquired using a Hitachi 7500 transmission electron microscope. The high contrast of the low-Z self-assembled material can possibly be due to the thermodynamically stable structure on the Formvar coating.

### 4.4. Circular Dichroism

Compounds CD spectroscopy was used for determining the chiral molecular arrangement of its supramolecular assemblies, in gelling (phosphate buffer). CD spectra were recorded between 170 and 600 nm using a Jasco J-810 spectrometer (Easton, MD, USA). Experiments were performed by placing the 1% gel material into a quartz cuvette of 1 mm path length and measurements were completed in triplicates. 

### 4.5. Rheology Studies

The viscoelastic properties of the material were studied and frequency sweep rheology experiments were performed to study the mechanical strength of the gels. Data was acquired using a Discover Hybrid Rheometer from TA instruments with a 20 mm, 1.997-degree cone, plate Peltier plate aluminum geometry and were completed in triplicate measurements. 

### 4.6. Enzyme Entrapment, Activity, Leaching, and Quantification Using Ultraviolet (UV) Scpectroscopy

50 μL of a 1 mg/20 mL solution of each enzyme was added into the gel solution upon cooling. Specifically, 10 mg of each compound was added to a vial with 1 mL of 50 mM monobasic phosphate buffer at pH 7. This solution was heated using a heat gun until all contents were dissolved and a clear solution was obtained. Upon cooling a hydrogel forms after roughly 30 min at this concentration. After 15 min of leaving the clear solution on the benchtop, where the solution is cooled to the touch but still in solution form, the enzyme solutions were added in and mixed thoroughly. After the addition of enzyme solution, we leave the vial on the bench to cool for longer and after roughly 10–15 min the hydrogel with the enzyme inside forms.

For enzyme leaching experiments, 2 mL of buffer was placed on top of the enzyme-entrapped hydrogels. 50 μL aliquots of buffer were taken out at various times points and were added to fresh substrate solution to see if the buffer aliquots could catalyze a reaction. The substrate solution for the HRP enzyme consists of 2.6 mL of 50 mM monobasic phosphate buffer pH 7, 0.16 mL of a 30% H_2_O_2_ solution, and 0.32 mL of a 50 mg/mL pyrogallol solution. The substrate solution for the amylase enzyme consists of 1 mL of a 100 mg/10 mL solution of starch, and 1 mL of 50 mM monobasic phosphate buffer pH 7. If the aliquots of buffer drawn up can catalyze a separate reaction, it is an indication that the enzyme has leached out of the gel and is just catalyzing the solution on top of the gel rather than from within the gel. The amount of activity can be traced back to how much enzyme leaches out. We have shown that there is not nearly as much activity from the aliquot drawn up, meaning the majority of the catalysis is within the gel. 

For quantification of activity, the substrate solutions for each enzyme were placed on top of the gel with the enzyme entrapped. The substrate solution on the HRP-entrapped gel was measured at 30 s, 1 min, 3 min, 5 min, 10 min, 1 h, and 24 h on the UV spectrophotometer at 420 nm, and the substrate solution on the amylase-entrapped gel was measured at 1, 3, 5, 10, and 30 min on the UV spectrophotometer at 540 nm. The products were then quantified using their respective extinction coefficients and the beer-lambert law.

For recycling and stability experiments, enzymes were entrapped in both hydrogels and substrate solution was placed on top of the gel every day for a week. The substrate solutions were quantified after 10 min on the gel each day to observe the activity of the enzyme over time. In addition, the enzyme was placed in a hydrogel and was left on the bench top at room temperature (about 21 degrees) for one month, after this time substrate solution was placed on top to see if the enzyme was still active by measuring the solution on top of the gel using the UV spectrophotometer at each respective wavelength and extinction coefficient to quantify.

## 5. Conclusions

In this study, two hydrogels were synthesized, characterized, and explored as an optimal matrix for enzyme entrapment. It was found that these hydrogels are extremely stable over time, retaining their viscoelasticity properties for one year. Besides the different chiralities, both compounds exhibited similar morphologies, strength, and abilities to entrap enzymes while retaining activity. Both compounds were able to entrap two enzymes—horseradish peroxidase and α-amylase—and the quantification of the product over time was performed. The ability of the enzyme to retain activity for long periods of time and for the enzyme biocatalyst to be reused after multiple days on a new substrate solution was also shown. These materials have great potential and show promise to advance the use of enzymes as biocatalysts on industrial scales for greener, more selective, and cheaper chemical production.

## Figures and Tables

**Table 1:** MGC, T_gel_, and Tan δ (all at 1%) values of hydrogels in 50 mM monobasic phosphate buffer at pH 7.

Compound	MGC (*w/v*)	T_gel_ (°C) (MGC/1%)	Tan δ (Gel-)	Tan δ (Gel + HRP)	Tan δ (Gel + Amylase)
MA-l-Phe-OH	0.25%	37/51	0.41 ± 0.03	0.43 ± 0.07	0.42 ± 0.12
MA-d-Phe-OH	0.25%	37/51	0.41 ± 0.11	0.43 ± 0.02	0.42 ± 0.07

## Supplementary Materials

Figure S1. ^1^H-NMR spectrum of MA-l-Phe-OH. Figure S2. ^13^C-NMR spectrum of MA-l-Phe-OH. Figure S3. ESI-MS spectrum of MA-l-Phe-OH. Figure S4. FTIR spectrum of MA-l-Phe-OH. Figure S5. ^1^H-NMR spectrum of MA-d-Phe-OH. Figure S6. ^13^C-NMR spectrum of MA-d-Phe-OH. Figure S7. ESI-MS spectrum of MA-d-Phe-OH. Figure S8. FTIR spectrum of MA-d-Phe-OH. Figure S9. Histograms of TEM fiber thickness for (a) MA-l-Phe-OH and (b) MA-d-Phe-OH. Figure S10. Additional TEM images of both hydrogels with entrapped enzymes. (a–c) M-l-Phe-OH hydrogel with HRP enzyme entrapped. Scale bar is 2, 2, and 1 m respectively. (d–f) M-l-Phe-OH hydrogel with amylase enzyme entrapped. Scale bar is 2, 1, and 1 m respectively. (g–i) M-d-Phe-OH hydrogel with HRP enzyme entrapped. Scale bar is 2 m for all. (j–l) M-d-Phe-OH hydrogel with amylase enzyme entrapped. Scale bar is 2 m for all. Figure S11. Rheology of hydrogels with entrapped enzyme. (a) MA-l-Phe-OH + HRP. (b) MA-d-Phe-OH + HRP. (c) MA-l-Phe-OH + amylase. (d) MA-d-Phe-OH + amylase. Figure S12. Time versus absorbance plot of purpurogallin product formation at 420 nm using the HRP enzyme in solution. Due to the addition of color reagent needed to detect the maltose product, the color change to red indicates the active free amylase enzyme in solution. Figure S13. Standard concentration versus absorbance spectrum maltose with color reagent for determination of extinction coefficient. Figure S14. Enzyme leaching experiment. Here the activity of the enzyme in the gel with substrate solution placed on the gel is compared to the activity of the catalytic reactions using the buffer solution on the enzyme in gel as the enzyme source. Here it is determined whether the activity comes from the enzyme leaching out the gel and catalyzing the reaction in the solution on top of the gel or the catalytic reaction occurring through the gel. (a) Activity comparison of HRP enzyme in MA-l-Phe-OH hydrogel with substrate solution on top (dark blue) versus activity of catalytic reaction using the enzyme leached out of gel in solely buffer on top of the gel as the enzyme source (light blue). (b) Activity comparison of HRP enzyme in MA-d-Phe-OH hydrogel with substrate solution on top (dark blue) versus activity of catalytic reaction using the enzyme leached out of gel in solely buffer on top of the gel as the enzyme source (light blue). (c) Activity comparison of amylase enzyme in MA-l-Phe-OH hydrogel with substrate solution on top (dark blue) versus activity of catalytic reaction using the enzyme leached out of gel in solely buffer on top of the gel as the enzyme source (light blue). (d) Activity comparison of amylase enzyme in MA-d-Phe-OH hydrogel with substrate solution on top (dark blue) versus activity of catalytic reaction using the enzyme leached out of gel in solely buffer on top of the gel as the enzyme source (light blue). Figure S15. (a) Image of two L followed by the last two vials of D hydrogels, the first and third vial containing the HRP enzyme and one does not. The substrate solution is placed on top all four vials while the HRP enzyme was added to the second and fourth solution (the vials without HRP entrapped). A color change in the hydrogels is observed for the gels with enzyme entrapped as substrate solution diffuses in and the colored product is formed. For the hydrogels that do not have the enzyme entrapped in the gel no color change is seen in the gel, only in the substrate solution. (b) Closer view of the vials with and without the enzyme entrapped. Figure S16. Stability test of enzymes in the gel for one month. (a) Comparison of activity of HRP enzyme in MA-l-Phe-OH hydrogel on day 1 to day 30. (b) Comparison of activity of HRP enzyme in MA-d-Phe-OH hydrogel on day 1 to day 30. (c) Comparison of activity of amylase enzyme in MA-l-Phe-OH hydrogel on day 1 to day 30. (d) Comparison of activity of amylase enzyme in MA-d-Phe-OH hydrogel on day 1 to day 30.
