# Supplementary material for: Enzyme Entrapment in Amphiphilic Myristyl-Phenylalanine Hydrogels
Source: Molecules. 2019 Aug 8;24(16):2884. doi: 10.3390/molecules24162884 (PMC6721053; doi:10.3390/molecules24162884)
Supplement: Supplementary file 1 [file molecules-24-02884-s001.pdf]

Supplementary Materials

Enzyme Entrapment in Amphiphilic Myristyl-Phenylalanine Hydrogels

Natashya Falcone [1,2], Tsuimy Shao [2,3], Roomina Rashid [2], and Heinz-Bernhard Kraatz [1,2,3*]

1 Department of Chemical Engineering and Applied Chemistry, University of Toronto, 200 College Street, M5S 3E5, Toronto, Canada
2 Department of Physical and Environmental Sciences, University of Toronto Scarborough, 1065 Military Trail, M1C 1A4, Scarborough, Canada
3 Department of Chemistry, University of Toronto, 80 St. George Street, M5S 3H6, Toronto, Canada
* Correspondence: Bernie.kraatz@utoronto.ca; Tel.: 416-287-7197

Figure S1. ^{1}H-NMR spectrum of MA-L-Phe-OH.
Figure S2. ^{13}C-NMR spectrum of MA-L-Phe-OH.
Figure S3. ESI-MS spectrum of MA-L-Phe-OH.
Figure S4. FTIR spectrum of MA-L-Phe-OH.
Figure S5. ^{1}H-NMR spectrum of MA-D-Phe-OH.
Figure S6. ^{13}C-NMR spectrum of MA-D-Phe-OH.
Figure S7. ESI-MS spectrum of MA-D-Phe-OH.
Figure S8. FTIR spectrum of MA-D-Phe-OH.
Figure S9. Histograms of TEM fiber thickness for **a**) MA-L-Phe-OH and **b**) MA-D-Phe-OH.
Figure S10. Additional TEM images of both hydrogels with entrapped enzymes. **a–c**) M-L-Phe-OH hydrogel with HRP enzyme entrapped. Scale bar is 2, 2, and 1 m respectively. **d–f**) M-L-Phe-OH hydrogel with amylase enzyme entrapped. Scale bar is 2, 1, and 1 m respectively. **g–i**) M-D-Phe-OH hydrogel with HRP enzyme entrapped. Scale bar is 2 m for all. **j–l**) M-D-Phe-OH hydrogel with amylase enzyme entrapped. Scale bar is 2 m for all.
Figure S11. Rheology of hydrogels with entrapped enzyme. **a**) MA-L-Phe-OH + HRP. **b**) MA-D-Phe-OH + HRP. **c**) MA-L-Phe-OH + amylase. **d**) MA-D-Phe-OH + amylase.
Figure S12. Time versus absorbance plot of purpurogallin product formation at 420 nm using the HRP enzyme in solution. Due to the addition of color reagent needed to detect the maltose product, the color change to red indicates the active free amylase enzyme in solution.
Figure S13. Standard concentration versus absorbance spectrum maltose with color reagent for determination of extinction coefficient.
Figure S14. Enzyme leaching experiment. Here the activity of the enzyme in the gel with substrate solution placed on the gel is compared to the activity of the catalytic reactions using the buffer solution on the enzyme in gel as the enzyme source. Here it is determined whether the activity comes from the enzyme leaching out the gel and catalyzing the reaction in the solution on top of the gel or the catalytic reaction occurring through the gel. **a**) Activity comparison of HRP enzyme in MA-L-Phe-OH hydrogel with substrate solution on top (dark blue) versus activity of catalytic reaction using the enzyme leached out of gel in solely buffer on top of the gel as the enzyme source (light blue). **b**) Activity comparison of HRP enzyme in MA-D-Phe-OH hydrogel with substrate solution on top (dark blue) versus activity of catalytic reaction using the enzyme leached out of gel in solely buffer on top of the gel as the enzyme source (light blue). **c**) Activity comparison of amylase enzyme in MA-L-Phe-OH hydrogel with substrate solution on top (dark blue) versus activity of catalytic reaction using the enzyme leached out of gel in solely buffer on top of the gel as the enzyme source (light blue). **d**) Activity comparison of amylase enzyme in MA-D-Phe-OH

hydrogel with substrate solution on top (dark blue) versus activity of catalytic reaction using the enzyme leached out of gel in solely buffer on top of the gel as the enzyme source (light blue).

Figure S15. **a**) Image of two L followed by the last two vials of D hydrogels, the first and third vial containing the HRP enzyme and one does not. The substrate solution is placed on top all four vials while the HRP enzyme was added to the second and fourth solution (the vials without HRP entrapped). A color change in the hydrogels is observed for the gels with enzyme entrapped as substrate solution diffuses in and the colored product is formed. For the hydrogels that do not have the enzyme entrapped in the gel no color change is seen in the gel, only in the substrate solution. **b**) Closer view of the vials with and without the enzyme entrapped.

Figure S16. Stability test of enzymes in the gel for one month. **a**) Comparison of activity of HRP enzyme in MA-L-Phe-OH hydrogel on day 1 to day 30. **b**) Comparison of activity of HRP enzyme in MA-D-Phe-OH hydrogel on day 1 to day 30. **c**) Comparison of activity of amylase enzyme in MA-L-Phe-OH hydrogel on day 1 to day 30. **d**) Comparison of activity of amylase enzyme in MA-D-Phe-OH hydrogel on day 1 to day 30.

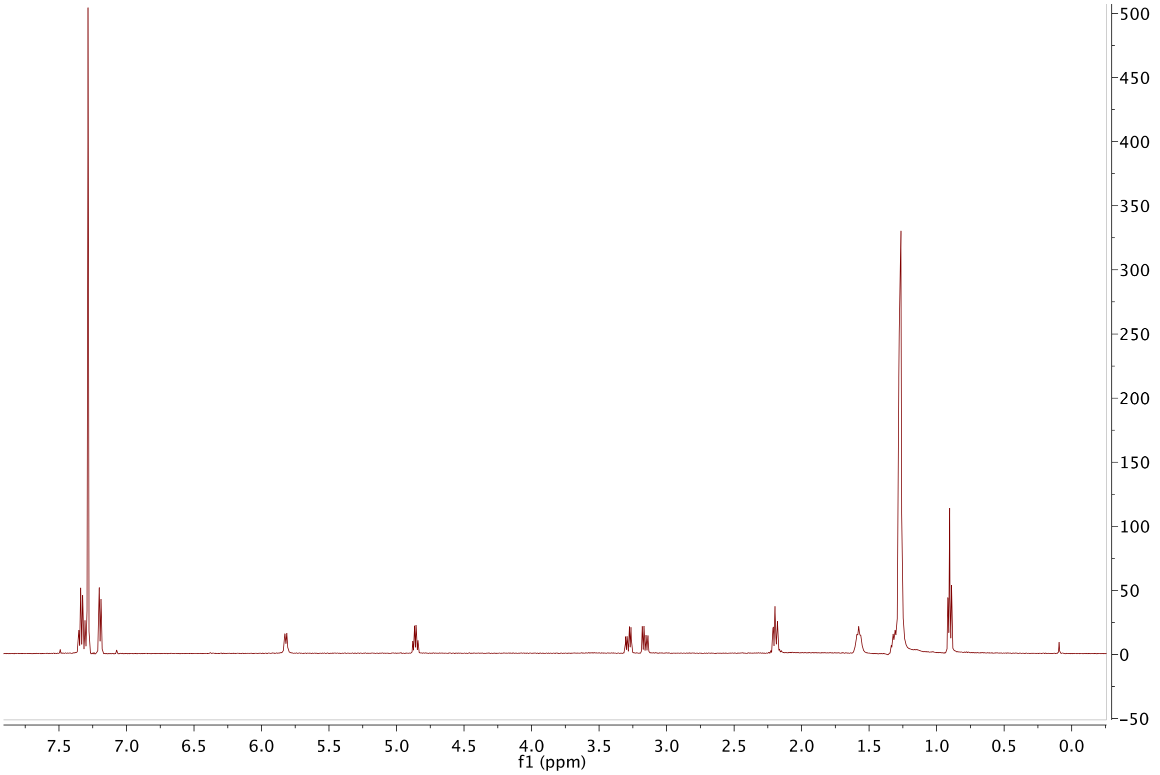

Figure S1. ^{1}H-NMR spectrum of MA-L-Phe-OH.

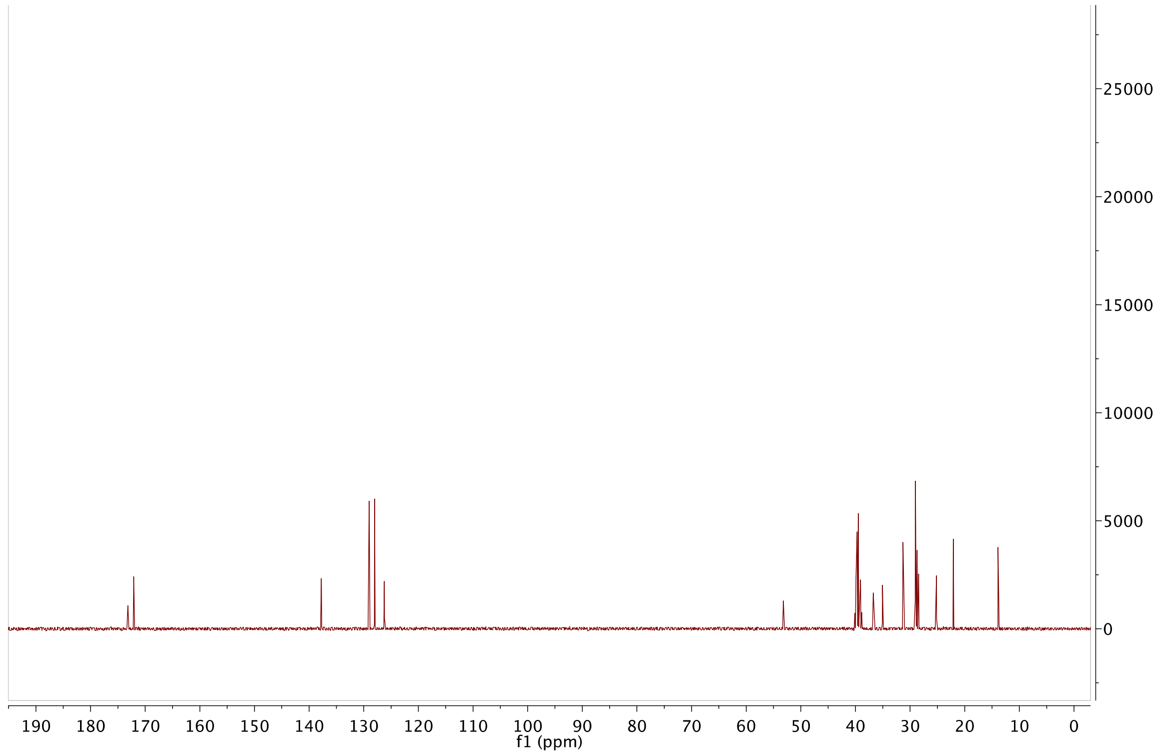

Figure S2. ^{13}C-NMR spectrum of MA-L-Phe-OH.

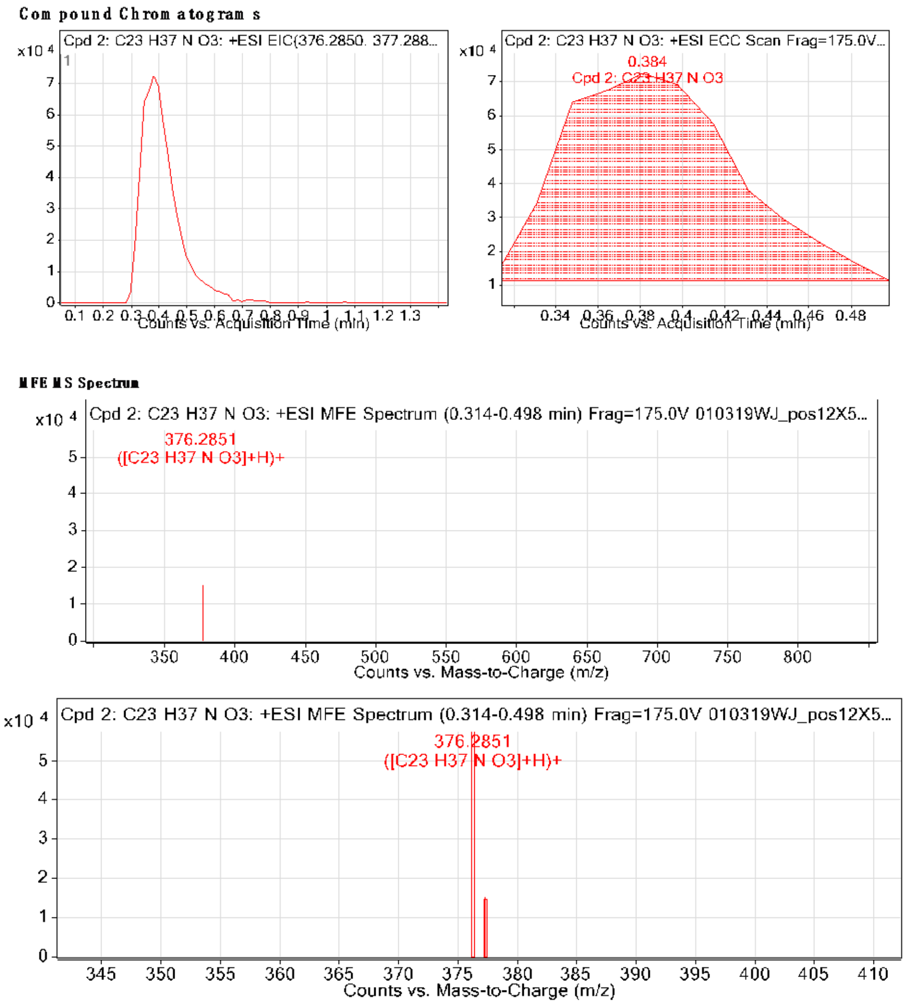

Figure S3. ESI-MS spectrum of MA-L-Phe-OH.

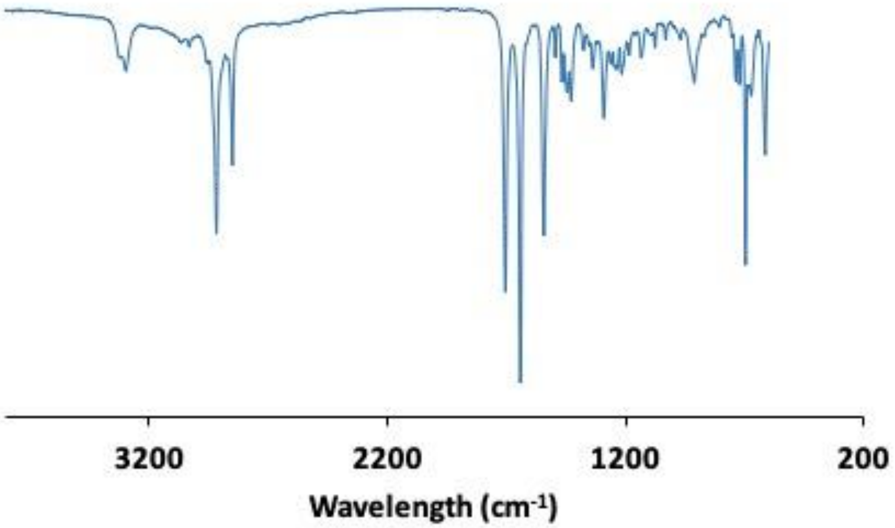

Figure S4. FTIR spectrum of MA-L-Phe-OH.

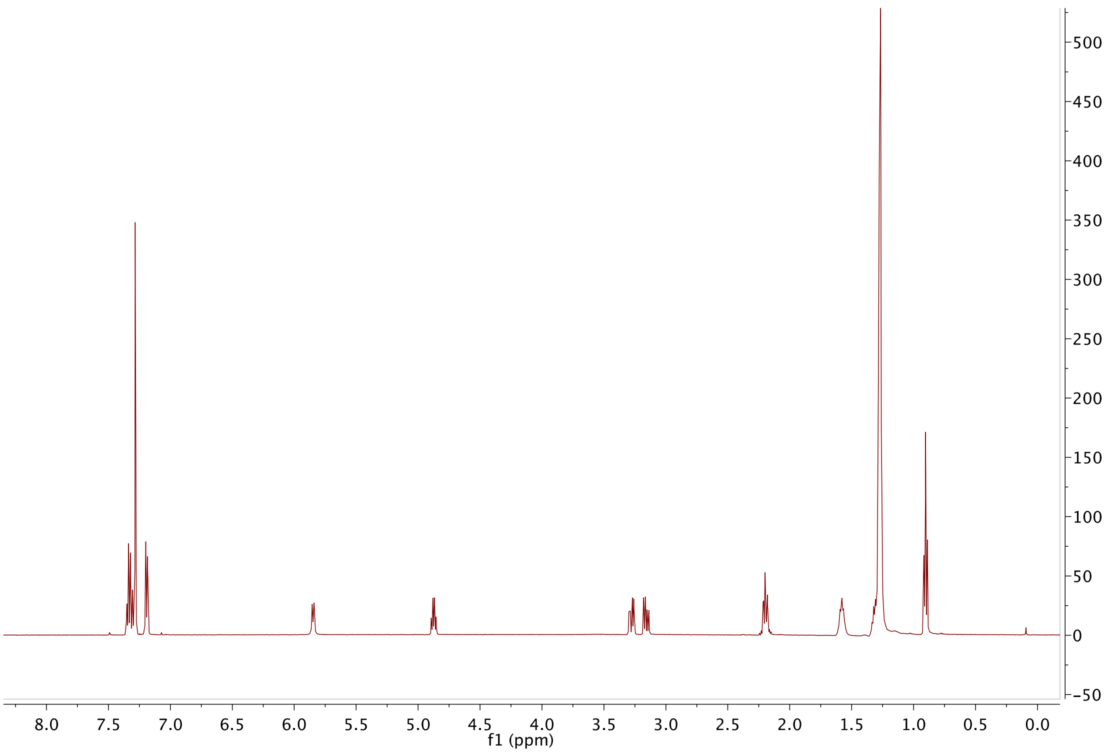

Figure S5. ^{1}H-NMR spectrum of MA-D-Phe-OH.

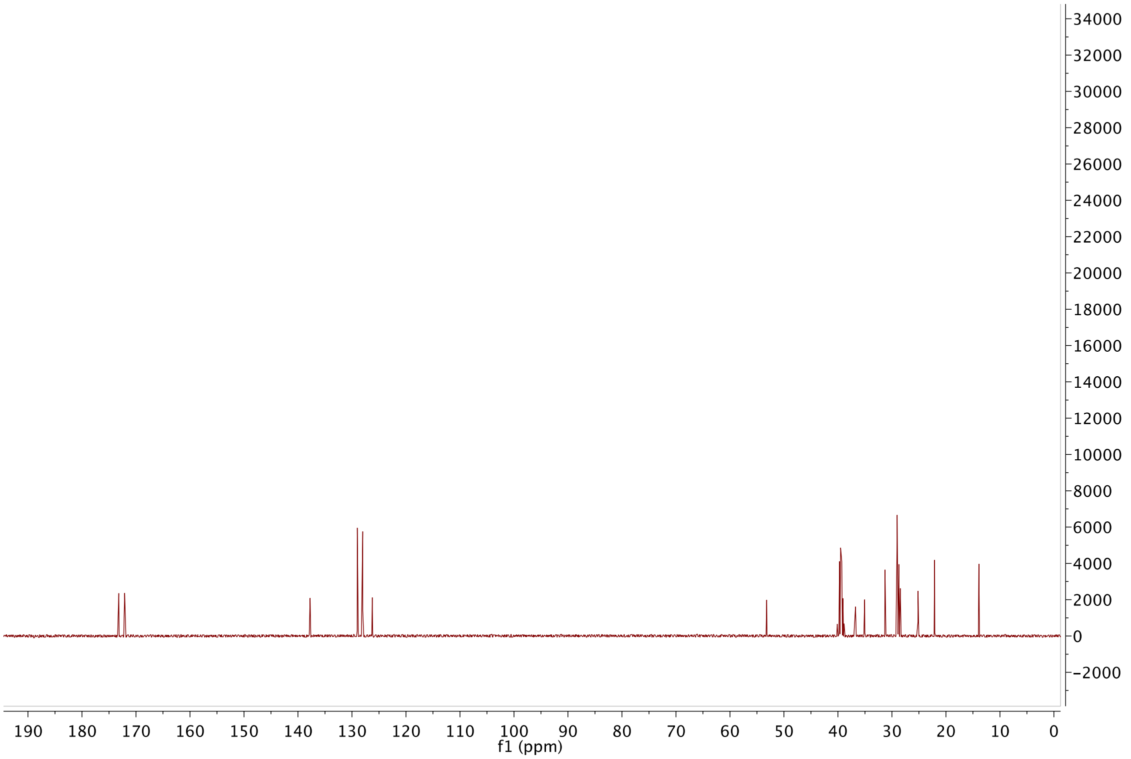

Figure S6. ^{13}C-NMR spectrum of MA-D-Phe-OH

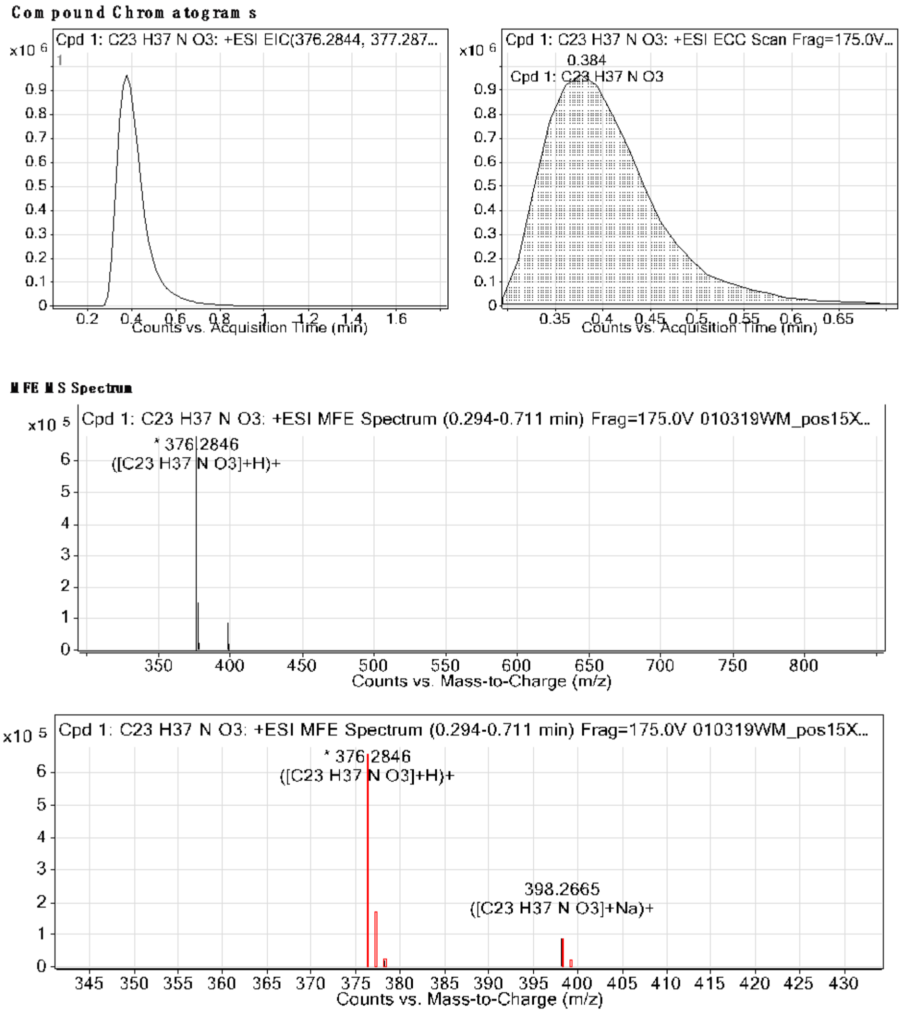

Figure S7. ESI-MS spectrum of MA-D-Phe-OH.

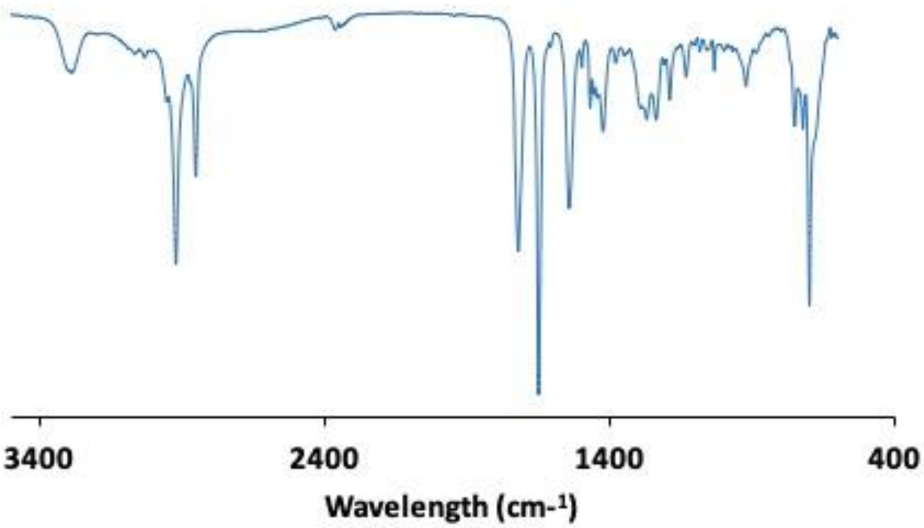

Figure S8. FTIR spectrum of MA-D-Phe-OH.

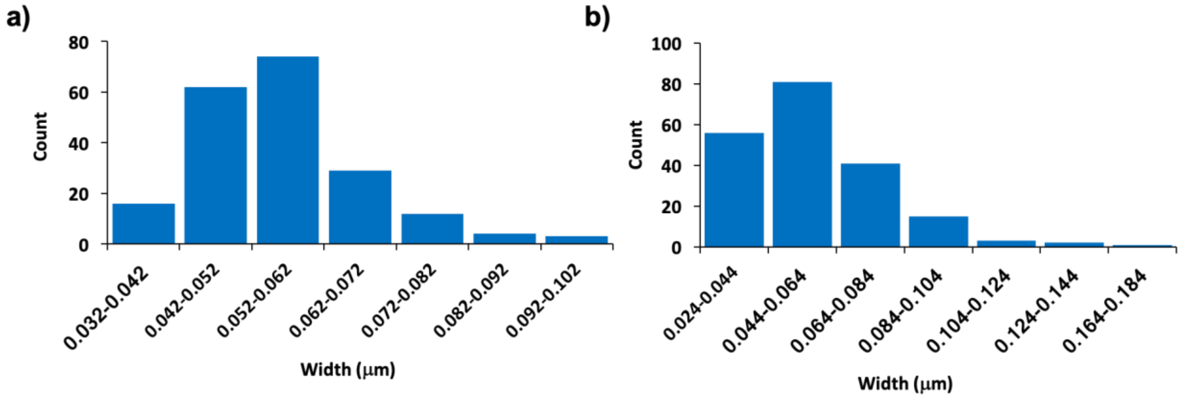

Figure S9. Histograms of TEM fiber thickness for **a**) MA-L-Phe-OH and **b**) MA-D-Phe-OH.

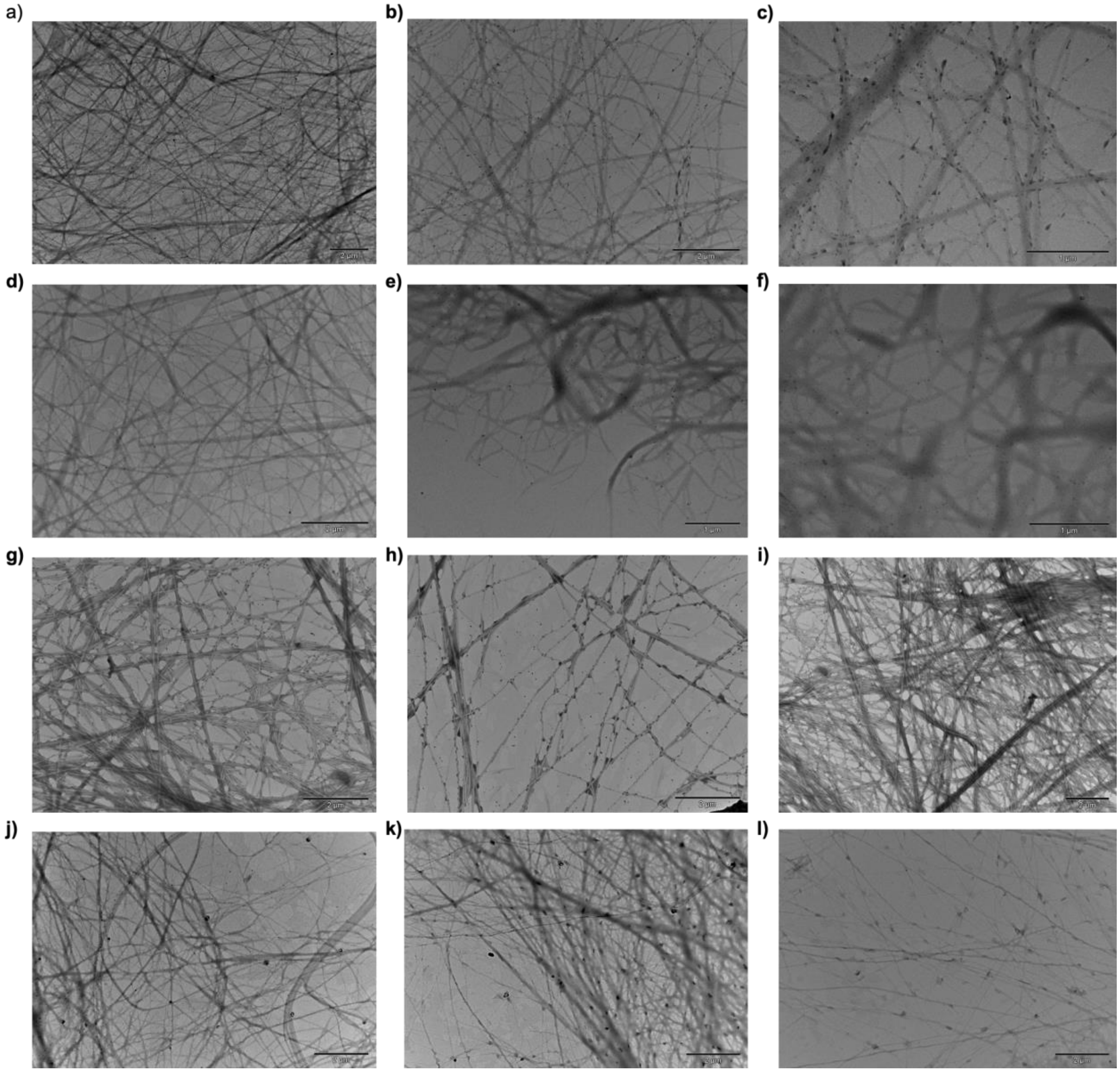

Figure S10. Additional TEM images of both hydrogels with entrapped enzymes. **a–c**) M-L-Phe-OH hydrogel with HRP enzyme entrapped. Scale bar is 2, 2, and 1 m respectively. **d–f**) M-L-Phe-OH hydrogel with amylase enzyme entrapped. Scale bar is 2, 1, and 1 m respectively. **g–i**) M-D-Phe-OH hydrogel with

HRP enzyme entrapped. Scale bar is 2 m for all. **j–l**) M-D-Phe-OH hydrogel with amylase enzyme entrapped. Scale bar is 2 m for all.

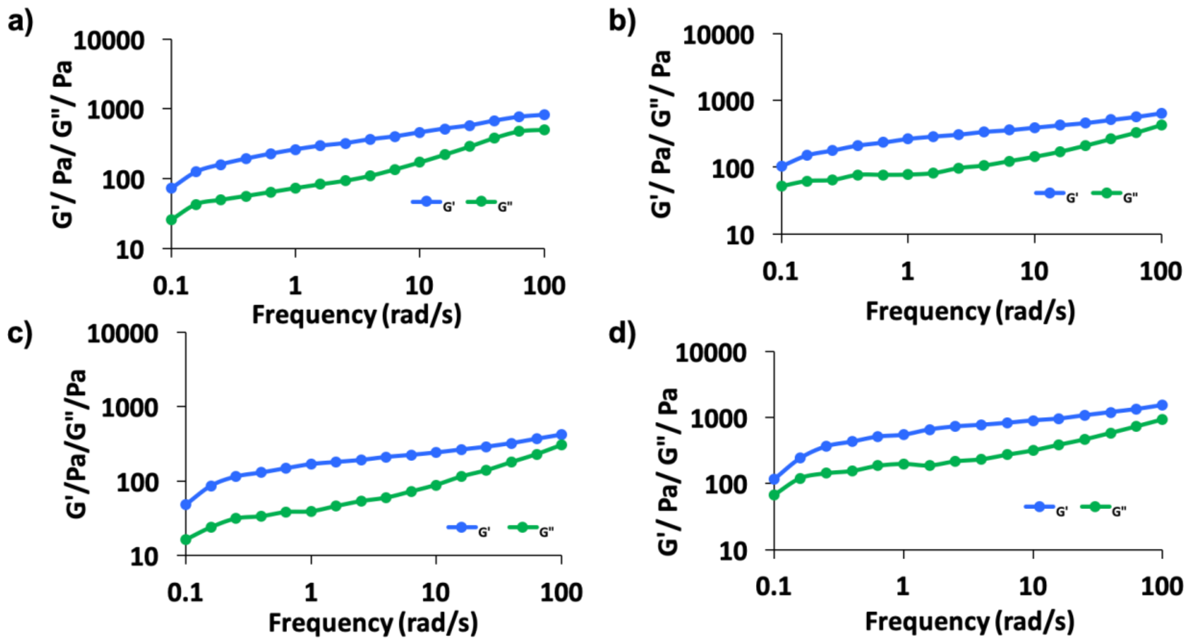

Figure S11. Rheology of hydrogels with entrapped enzyme. **a**) MA-L-Phe-OH + HRP. **b**) MA-D-Phe-OH + HRP. **c**) MA-L-Phe-OH + amylase. **d**) MA-D-Phe-OH + amylase.

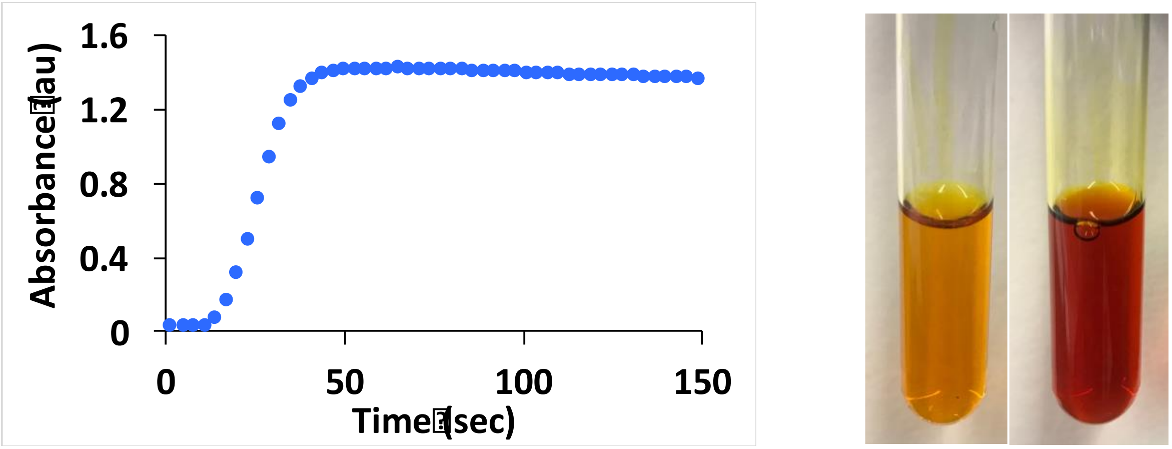

Figure S12. Time versus absorbance plot of purpurogallin product formation at 420 nm using the HRP enzyme in solution. Due to the addition of the color reagent needed to detect the maltose product, the color change to red indicates the active free amylase enzyme in solution.

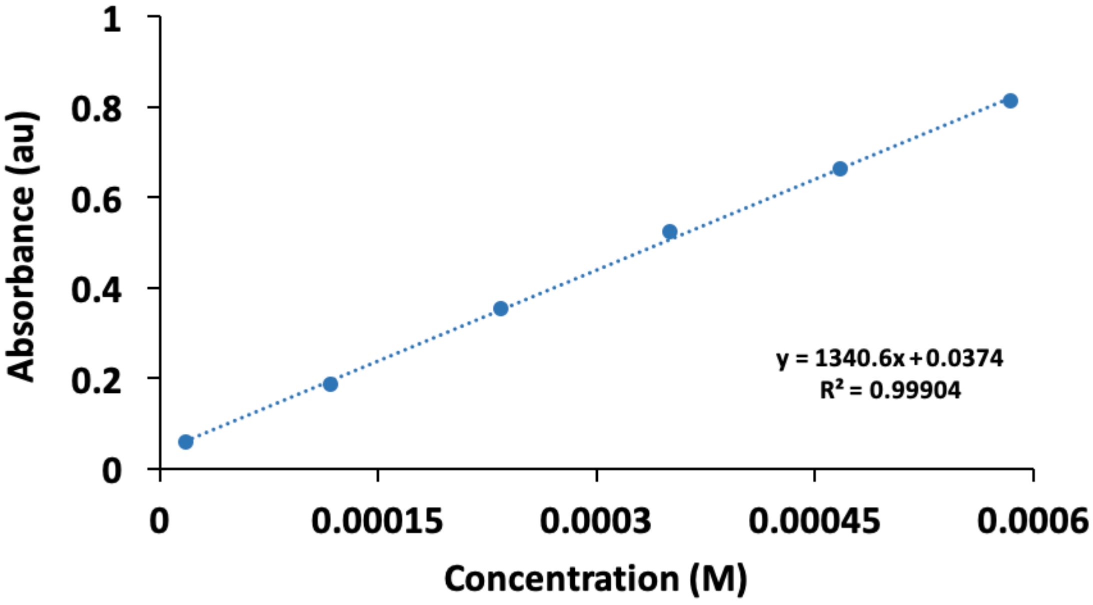

Figure S13. Standard concentration versus absorbance spectrum maltose with the color reagent for determination of extinction coefficient.

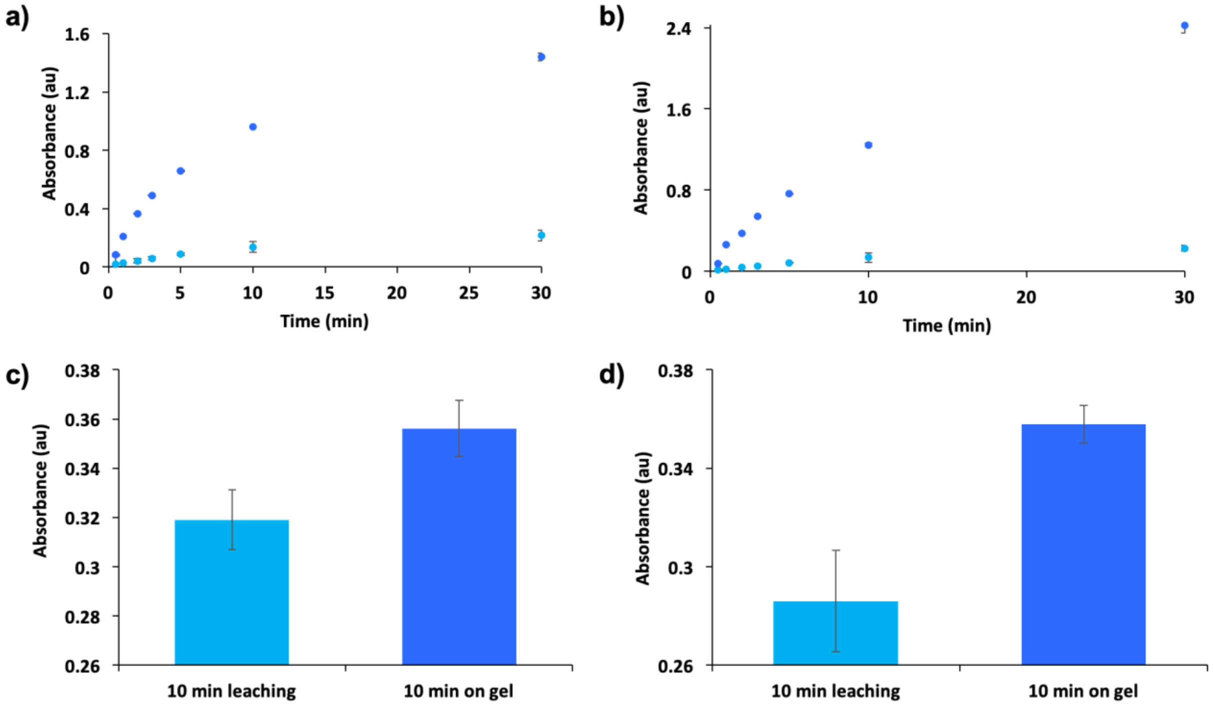

Figure S14. Enzyme leaching experiment. Here the activity of the enzyme in the gel with the substrate solution placed on the gel is compared to the activity of the catalytic reactions using the buffer solution on the enzyme in gel as the enzyme source. Here it is determined whether the activity comes from the enzyme leaching out the gel and catalyzing the reaction in the solution on top of the gel or the catalytic reaction occurring through the gel. **a**) Activity comparison of HRP enzyme in MA-L-Phe-OH hydrogel with

substrate solution on top (dark blue) versus activity of catalytic reaction using the enzyme leached out of gel in solely buffer on top of the gel as the enzyme source (light blue). **b**) Activity comparison of HRP enzyme in MA-D-Phe-OH hydrogel with substrate solution on top (dark blue) versus activity of catalytic reaction using the enzyme leached out of the gel in solely the buffer on top of the gel as the enzyme source (light blue). **c**) Activity comparison of amylase enzyme in MA-L-Phe-OH hydrogel with substrate solution on top (dark blue) versus activity of catalytic reaction using the enzyme leached out of gel in solely buffer on top of the gel as the enzyme source (light blue). **d**) Activity comparison of amylase enzyme in MA-D-Phe-OH hydrogel with substrate solution on top (dark blue) versus activity of catalytic reaction using the enzyme leached out of the gel in solely the buffer on top of the gel as the enzyme source (light blue).

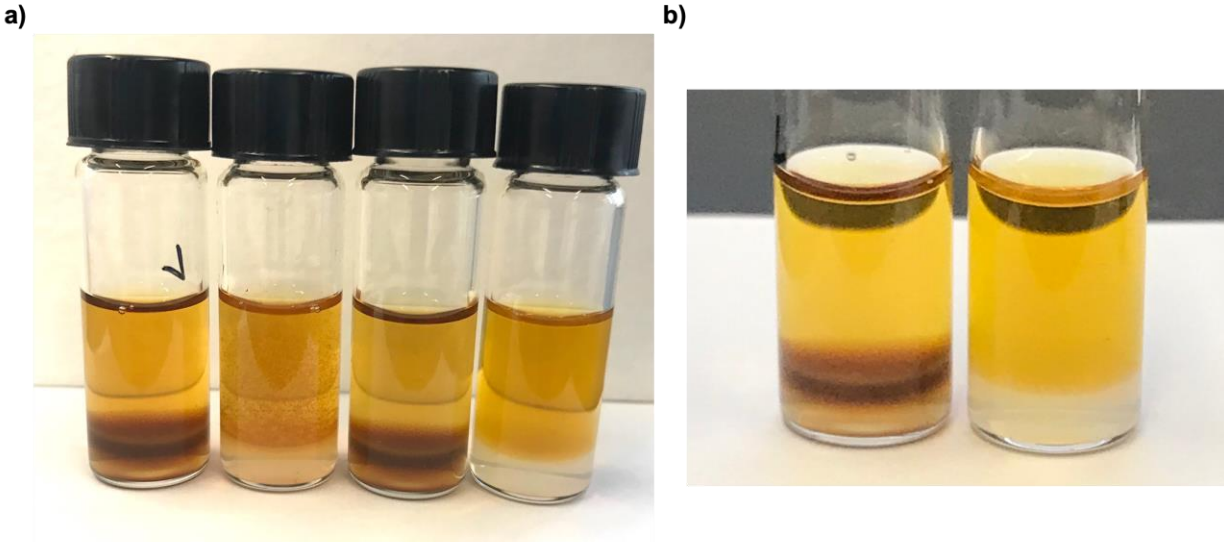

Figure S15. **a**) Image of two L followed by the last two vials of D hydrogels, the first and third vial containing the HRP enzyme and one does not. The substrate solution is placed on top all four vials while the HRP enzyme was added to the second and fourth solution (the vials without HRP entrapped). A color change in the hydrogels is observed for the gels with enzyme entrapped as substrate solution diffuses in and the colored product is formed. For the hydrogels that do not have the enzyme entrapped in the gel no color change is seen in the gel, only in the substrate solution. **b**) Closer view of the vials with and without the enzyme entrapped.

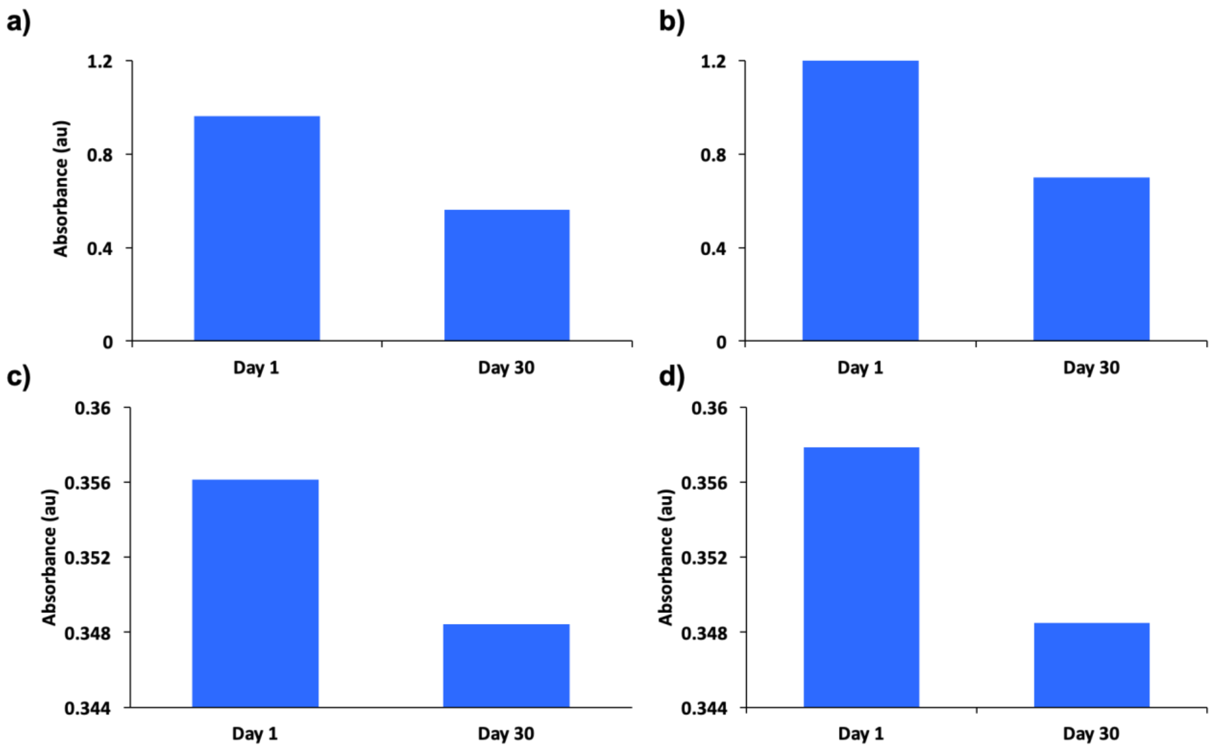

Figure S16. Stability test of enzymes in the gel for one month. **a**) Comparison of activity of HRP enzyme in MA-L-Phe-OH hydrogel on day 1 to day 30. **b**) Comparison of activity of HRP enzyme in MA-D-Phe-OH hydrogel on day 1 to day 30. **c**) Comparison of activity of amylase enzyme in MA-L-Phe-OH hydrogel on day 1 to day 30. **d**) Comparison of activity of amylase enzyme in MA-D-Phe-OH hydrogel on day 1 to day 30.
